# Implementing screening programmes in primary care versus a centralised administration: a qualitative study of atrial fibrillation screening

**DOI:** 10.1186/s12875-026-03172-1

**Published:** 2026-01-20

**Authors:** Rakesh N Modi, Jonathan Mant, Kate Williams, Andrew Dymond, Jenni Burt

**Affiliations:** 1 Primary Care Unit, Department of Public Health and Primary Care, University of Cambridge, 2 Worts’ Causeway, Cambridge, CB1 8RN UK; 2 THIS Labs, Office 308, Regus, Chivers Way, Cambridge, CB24 9AD UK

**Keywords:** Implementation, Mass screening, Primary care, Public health, Atrial fibrillation

## Abstract

**Background:**

General practices have been tasked with increasing detection of atrial fibrillation (AF) to reduce stroke. Paroxysmal AF is often missed through usual care but can be detected through screening with repeated ECGs over a period of time using hand-held ECG devices. As part of the drive to detect AF, screening with such devices is being encouraged by both policy makers and industry. It is unclear whether general practice should be leading this effort. Previously, we showed that there was no quantitative difference between a centralised administration and general practice-delivered AF screening programme in terms of the quality and numbers of ECGs generated. Here, we aimed to assess the strengths and weaknesses of each approach using qualitative methods.

**Methods:**

We compared programme delivery by one UK general practice and by a non-clinical centralised administration for two UK general practices in a qualitative study to explore how to conduct screening for AF in a planned trial. From September to December 2020, we conducted semi-structured interviews with 19 staff members. We took field notes of implementation issues arising during observation of 4.5 h of training and collected 15 training evaluation forms. Data were analysed thematically sensitised by the Consolidated Framework for Implementation Research. Analysis focused on the strengths and weaknesses of the different approaches.

**Results:**

While both general practice staff and centralised administrators showed motivation to deliver a screening programme, there were differences in skills and capacity. General practice staff provided continuity of care and offer other care in parallel. They could use relational and communication skills to potentially engage those from underserved communities, but were limited by resources. Centralised administrators, with a singular focus on screening, could deliver a consistently high performance and undertake more complicated administration. Their initial anxieties about communication skills reduce with training and experience.

**Conclusions:**

In screening for AF, primary care and centralised administration demonstrate different strengths and weaknesses. A hybrid approach with centralised screening and primary care signposting, particularly for underserved communities, might be optimal. Awareness of this may help policy makers optimise the use of primary care in the drive to detect AF.

**Supplementary Information:**

The online version contains supplementary material available at 10.1186/s12875-026-03172-1.

## Introduction

National and international health policy-making bodies prioritise the detection of atrial fibrillation (AF) to reduce strokes [1]. Although it is not yet clear whether systematic screening for AF is worthwhile, opportunistic screening is becoming increasingly encouraged by these health bodies. Hand-held ECG devices can detect undiagnosed paroxysmal AF through intermittent screening at home and general practices are being encouraged to use them. However, as general practice is already under significant pressure, an alternative model of whether screening for AF at home without the use of primary care might be attractive.

Screening programmes are often delivered largely by primary care, a centralised non-clinical administration or a hybrid of both. For example, in the UK, a primary care-led model is used in routine physical health checks of 6- to 8-week old babies and their mothers[2]; a remote and centralised model is used in bowel cancer screening[3]; and with invitations sent centrally, and testing delivered in primary care, a hybrid model is used for cervical sceening[4]. It is uncommon to compare the differences between primary care and a centralised administration for the delivery of the same screening programme.

**Fig. 1:**
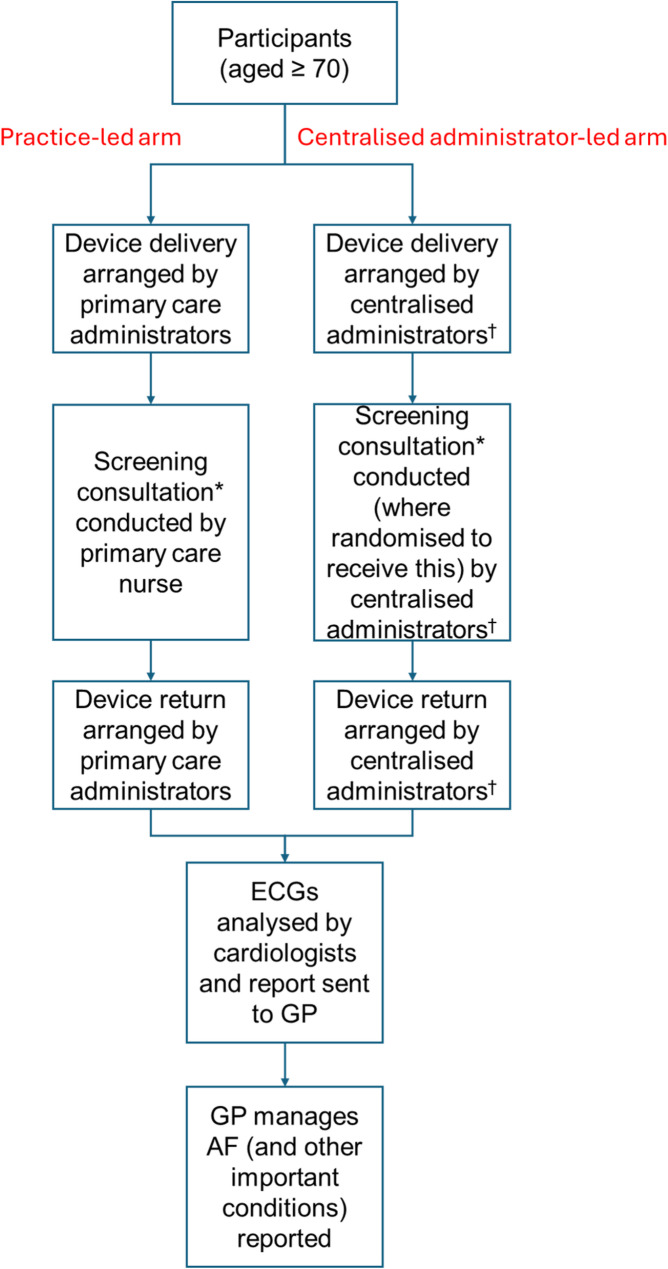
Practice-led and centralised administrator-led arms of a SAFER feasibility study. ECG: Electrocardiogram; GP: general practitioner; AF: atrial fibrillation. *a screening consultation is a call of approximately 10 min made to the participant after they have received the device to explain the use of the device, answer any questions, refine the participant’s technique until they can produce a test ECG of reasonable quality, and explain how to return the device. Staff were supported in this by a checklist (see Additional File 1). ^†^the centralised administrators were the SAFER study administrators based in the University of Cambridge

We had the opportunity to study these differences in a feasibility study ahead of the main SAFER trial, which is evaluating the effectiveness and cost-effectiveness of screening for AF with a portable (mobile signal-reliant) hand-held ECG device [5]. This feasibility study was required as a result of the COVID pandemic, which necessitated a switch from in person to remote screening. Participants were assigned to have screening led either by the staff at their general practice or led by a centralised administration (the SAFER study administrators) (see Fig. 1). In the centralised administration arm, participants were randomised to receive various levels of support.

We have previously reported data from this feasibility study to show that remote screening achieved the same uptake as face-to-face screening (87.5% vs. 90.0%, *p* = 0.198) with similar ECG quality and quantity (97.2% vs. 98.3% of participants produced ≥ 56 interpretable ECG traces, *p*= 0.257)[6]. We also found that there were no important differences in the quality and quantity of ECGs when comparing remote training by a central administrator to remote training by general practice (98.8% vs. 100% of participants produced ≥ 56 interpretable ECG traces, *p*= 0.39)[7]. However, nuances in the advantages and disadvantages between general practice and centralised administration were not explored. Here, we report qualitative data on the strengths and weaknesses of primary care and centralised administration in delivering screening programmes for atrial fibrillation.

## Methods

### Design

This was a multi-method qualitative study embedded within a SAFER feasibility study.

### Setting

The setting, approach to screening and arms are described in our previous paper [7]. In short, the study took place between October and December 2020 in three general practices (Practices 1, 2 and 3) in the East of England identified by the local National Institute for Health and Care Research (NIHR) Clinical Research Network (CRN). This was during the Covid-19 pandemic when workloads increased and have since remained high [8]. The practices were medium sized, ranging from 7,000 to 12,000 registered patients and included two rural (practices 2 and 3) and one urban (practice 1) practice. In terms of deprivation, they were based in the 5th (practice 1), 7th (practice 3) and 10th (practice 2) least deprived deciles according to the Index of Multiple Deprivation. The proportion of participants with AF and a CHADS_2_DS_2_VASc score (a scoring system with higher scores reflecting a higher risk of stroke in AF) [9] of 2 or more (indicating that anticoagulation should ordinarily be prescribed) [10] who were prescribed anticoagulation varied from 86.1 to 93.1% (median for England was 90.6%)[11]. The practices were a convenience sample selected due to ability to deliver a feasibility study and identified by the local Clinical Research Network, who hold a database of research active practices.

### Intervention

Participants from Practice 1 were allocated to a practice-led arm, and participants from Practices 2 and 3 were allocated to a centralised administrator-led arm. The intervention in both arms is described in Fig. 1. The checklist to support staff to conduct the screening consultation is shown in Additional file 1.

### Data collection

RM, a General Practitioner (GP) partner and researcher from the East of England collected and led analysis of all data. An overview of the specific methods, samples and dates of data collected are shown in Table 1.

**Table 1:** Methods of data collection

Method	Sample	Dates collected
Practice staff interviews	GPs, nurses and administrators from all practices	November – December 2020
Centralised administrator interviews	All centralised administrators	November – December 2020
Observations of training	All Site Initiation Visits (SIVs), screening training and centralised administrator training	September – November 2020
Training evaluation forms	All attendees of the observed training	September - November 2020

*SIV* Site Initiation Visit

#### Interviews

We conducted semi-structured, in-depth, telephone or video mediated, and audio-recorded interviews lasting 25–74 min with all centralised administrator staff (*n* = 5) and a purposive selection of practice staff (*n* = 14). For practice staff, the research administrator in each practice was asked to provide contact details for staff in various roles including GPs, nurses, administrators, research staff and the practice manager. Practice staff were selected for maximum variation in roles to explore all aspects of the screening programme. Only one round of selection of staff was required as it provided sufficient information power [12]. Information power is a conceptual sampling approach that can be used in qualitative studies to determine data sufficiency [12]. The characteristics of the interviewees are shown in Table 2.

**Table 2:** Characteristics of interviewees in a feasibility study of SAFER. Adapted from Modi 2023 [13].

Participant characteristic	*N*
Sex	
Female	15
Male	4
**Role**	
Centralised (study) administrators	5
Experienced senior staff	2
New senior staff	1
Experienced junior staff	1
New junior staff	1
Practice staff	14
GPs	4
Clinical nurses	2
Health care assistants	1
Practice managers	1
Receptionists	1
Research nurses	3*
Trial co-ordinators	2*
Junior research administrators	1
**Practice employing practice staff**	
Practice 1	6
Practice 2	2
Practice 3	6

* One trial co-ordinator was also a research nurse and therefore has been counted in both categories

We developed the topic guide to explore topics such as training required, fit with current work pressures, comfort with the role, and thoughts on large-scale implementation issues, sensitised by constructs in the Consolidated Framework for Implementation Research [14]. We then piloted and refined the topic guide through the first five interviews that included a variety of roles and both practice and centralised administrator staff (see final topic guide in Additional file 2).

After reading the Patient Information Sheet (PIS) and an Interview Consent Form (ICF), consent was signed by proxy by the interviewer at the start of the video/telephone call. Interviews were recorded on an encrypted audio-recorder, stored on a secure drive and audio files were transcribed verbatim by a professional transcription service. Anonymised analytical notes and a reflexive journal were electronically written after each interview [15]. [16]

#### Observations of training

RM undertook observations, taking in-depth field notes for all centralised administrator and practice staff training sessions focussing on implementation issues and differences between the teams. As with the interviews, he kept analytical notes and a reflexive journal. The method was piloted in two Site Initiation Visits (SIVs) that preceded this study, in February and March 2020. SIVs were held via video conference, which RM joined.

All training sessions were delivered by senior centralised administrator staff. For practice staff, they included three SIVs covering the background of the study (one to each of practices 1, 2 and 3 lasting one hour each), and a 1.5-hour screening training session for practice 1 on conducting screening consultations and delivering the device. Practice staff attendees were usually a research administrator, a GP and sometimes a nurse. Centralised administrator training consisted of three 1.5 h training sessions (total observation time of 4.5 h) for all junior or new team members covering the same topics.

Information on observations were provided in advance to trainers and attendees via email, and at the start of each training session, RM sought verbal consent to observe [16]. 

#### Training evaluation forms (TEFs)

We analysed the textual responses from all TEFs that were provided to all attendees at the training sessions.

With no previous such TEF in the literature, a new TEF was created based on the dominant Kirkpatrick model for evaluating training of healthcare professionals[17], WHO guidance[18], and the literature on best practice for questionnaires [19–21]. The TEFs were piloted during two SIVs predating the study, with textual responses assessed for adequacy by the research team.

TEFs were sent to attendees via email prior to training sessions. Time was provided at the start and end of training sessions to complete the form. They were returned to the researcher electronically. Consent to participate was assumed by completion and return of the TEFs [22]. We received 10 TEFs from practice staff and 5 from centralised administrators. Examples of TEFs are shown in Additional files 3 and 4.

### Analysis

We undertook a thematic analysis of data from all qualitative methods together, in line with Braun and Clarke’s method. This involved beginning the analysis after the first data collection event and continuing in an iterative manner throughout thereby helping to guide further data collection [23]. The approach to coding itself followed guidance form Saldana’s coding manual [15]. RM first familiarised himself with all data before coding text (transcripts or field notes) inductively (‘open coding’) and creating descriptive categories and then themes.

By revisiting codes and re-coding for meaning (‘thematic coding’), and iteratively re-working categories and themes, a more interpretive set of themes were created. As part of this, the Consolidated Framework for Implementatoin Research (CFIR) was ‘abducted’ by incorporating relevant barriers, enablers and strategies to implementation to refine the themes [14]. Finally, these were considered through the lens of the research question about the strengths and weaknesses of the various models to create a final set of relevant themes. Each of these themes were implementation features for which there were key differences between the practice staff and centralised administration models. The iterative steps were undertaken by RM and with regular analysis meetings with JB, a senior qualitative researcher, to look at data through a different perspective. This was aided by a reflexive journal and analytical notes that were also incorporated into the thematic analysis.

Codes, themes and categories were reviewed and refined through meetings amongst the researchers. Analysis was supported by NVivo V.12[24]. The final analysis was member checked with the centralised administrators via a poster presentation.

RM, who led the study, is an experienced GP and researcher with insights into usual practice and primary care burdens, as well as experience leading centralised teams. His experience-based views were made explicit and minimised through review of themes by the multi-disciplinary research team.

Ethical approval for the study and materials was obtained from the London-Central Research Ethics Committee (19/LO/1597). This report is consistent with the Standard for Reporting Qualitative Research (SRQR) guidelines (see Additional file 5).[25]

## Results

Our analysis identified specific features of the models or activities undertaken by the teams that were important for the delivery of this screening programme, and that were expressed or undertaken differently by practice staff or centralised administrators. These features that were different between the groups of staff became the themes of the analysis. The themes were: motivation, proficiency in consultation skills with participants, specialising and its impact on performance, desire to provide a positive experience for participants, consciousness of inequalities, availability of resources, scalability, and standalone versus joined-up care. We report our analysis under each.

### Motivation

Being motivated to deliver the AF screening programme was important for both sets of staff, but drivers of this motivation differed.

Practice staff relied on an inherent *“love [of] new projects”* (GP 1, practice 1, interview), and a need to *“get someone that it matters to or it excites them or they really like the patient contact”* (trial co-ordinator, practice 2, interview) for the programme to be successful. This emphasis on keen staff might be important in a resource constrained setting such as primary care.

Centralised administrators felt that having the “*right person”* who was self-motivated would help but also felt that *“with the proper training any role could do this”* (Experienced senior centralised administrator 2, interview).

Furthermore, in contrast to the practice team, the centralised administrators talked less about the inherent motivation of the staff member, and more about motivation coming from how they were managed. A personable style of management could be a motivator for them.

"…‘we’re really getting through these numbers so well done everyone.’ And, I don’t know, things like that; it doesn’t have to be massive gestures, little things can make a big difference."

(Experienced senior centralised administrator 1, interview)

### Proficiency in consultation skills with participants

Practice staff and centralised administrators differed in their strengths and weaknesses in consultation skills. We also identified differences between levels of seniority within the teams.

Practice staff overwhelmingly reported being comfortable in communicating with participants, including in conveying inherent uncertainty within screening results, and discussing sensitive topics.

"I think we are very much used to that, we know that these tests only give you a pinpoint, they don’t tell you at all what potentially might happen. I think we’re fairly used to explaining that to patients."

(GP2, practice 1, interview)

This contrasted with the centralised administrators, where junior members in particular, were concerned that *“certain language that can be taken incorrectly”* (new junior centralised administrator, centralised administrator training 1, TEF) or be *“confus[ing]”* (new junior centralised administrator, interview). Even senior centralised administrators were concerned, which became evident during the training sessions.

"[new senior centralised administrator] asked in a tone of curiosity, with regards to calls made due to poor quality traces, “how do you broach that?” [Experienced centralised administrator 1] answered… avoid blame and to avoid saying that anything is being done wrong… This topic of broaching poor quality [lasted] 5–10 min."

(centralised administrator training 3, observation field notes)

While centralised administrator training covered strategies to communicate about sensitive topics, junior members found the strategy of using scripts or checklists helpful to surmount any nervousness about consultations.

"[about having a script] Oh definitely much better having one. I’m not sure what I would have said otherwise. I probably would have drafted my own script, I wouldn’t have just winged it."

(New junior centralised administrator, interview)

"I don’t feel unprepared or concerned, just anxious in case I stutter on the phone or send the participant away with poor quality ECGs because I didn’t identify it during the test ECG."

(Experienced junior centralised administrator, centralised administrator training 3, TEF)

Another concern from junior non-clinicians was dealing with questions that were *“clinical in nature”* (research administrator, practice 1, interview). A strategy to deal with this was for them to couch the advice on the device in a disclaimer where they could *“reassure [the participant] that [the test ECG traces] are for reviewing technique only and not clinical appraisal”* (new senior centralised administrator, centralised administrator training 1, TEF). In contrast, the practice staff were either clinical themselves and could address any clinical issues or had the benefit of immediate access to relevant staff.

For junior centralised administrators, the need for checklists and fear of clinical questions were also resolved with experience and training as seniors *“*have the checklist there, but I don’t really refer to it a great deal anymore” (new senior centralised administrator, interview).

“That’s not to say that people can’t be trained through becoming more comfortable with that. Most people I think would be absolutely fine with it.”

(Experienced senior centralised administrator 2, interview)

It was unclear whether the improvements in consultation skills came from junior to senior staff, and non-clinical to clinical, came purely from experience, from training, or both.

### Specialising and its impact on performance

We identified when deciding between primary care and centralised administration a key consideration is use of staff that do screening work less often but have other patient care responsibilities, (primary care) or use of staff whose sole job is screening delivery (centralised administration). Unlike proficiency in consultation skills related to experience or clinical role (previous theme), this concerned narrowness of the tasks and, consequently, the performance level of that narrower set of tasks.

Centralised administrators had more experience than practice staff in dealing with complex administration related to the screening programme. This might have been because they had more time for training and development of detailed *“SOPs [Standard Operating Procedures] prepared in advance”* (new senior centralised administrator, centralised administrator training 1, TEF). Training for primary care staff to this level might not have been possible due to workload pressures (discussed further under ‘availability of resources’). In line with the specialisation and clear protocols, centralised administrators also expected *“consistency”* (trial co-ordinator, practice 2, interview) in quality.

Consistent with the ease of quality control of a centralised administration over primary care, it was noted that practice staff, distributed widely over several independent organisations, might be harder to quality assure and train than centralised staff in a single organisation. The former would involve *“training an external group that you can’t really check up on…”* (new senior centralised administrator, interview).

We found that centralised administrators were more able to deal with technical issues and troubleshooting issues with the device or software because *“this is what they do*,* they’re potentially more likely to sort the problem out”* (research nurse, practice 2, interview). Although a basic understanding of troubleshooting was sought by practices, they felt unable to provide more technical care due to resource shortages. This was identified as a reason to have screening run by centralised administrators.

"I’d like to say that we could manage it in primary care, but realistically I don’t think we can in addition to everything else. I think it would need to be outsourced to somebody that could deal with those ad hoc queries as and when they arose."

(Clinical nurse 2, practice 3, interview)

### Desire to provide a positive experience for participants

Centralised administrators discussed for longer and in more depth the need to please participants and meet their expectations, as compared to practice staff. There was anxiety about the possibility of upsetting participants in the former group, and conversation about how to avoid this.

With respect to the concern about disappointing participants, this might have been a result of the different power dynamics between participants and their primary care provider, versus the centralised team. This sometimes led to the centralised administrators trying to be more flexible than practice staff.

"But I think also people kind of expect that, if it’s being delivered from a GP practice, is that they are very much going to be, right, this is your appointment, this is the day, bash, bash, bash… whereas we make it quite clear when we’re talking to them on the phone… we are not anything within your practice. So I think people then expect a bit more flexibility and it to be on their terms."

(New senior centralised administrator, interview)

This desire of centralised administrators to not inconvenience patients was corroborated by observations of the concerns raised during training.

"[the senior centralised administrators] spoke a little anxiously about sending out the device and the patient wanting to know exactly when it would arrive… [they] did not seem happy to allow the patient to wait for it."

(centralised administrator training 1, field notes)

### Consciousness of inequalities

In terms of inequalities, practice staff proactively raised concerns in interviews about health inequalities and suggested that they could engage participants from underserved backgrounds.

"…but what do you do about people that maybe their true focus in life is my money runs out on Wednesday and I’ve still go to eat Thursday/Friday, what is going to bother them to a) have this device, not just stick it on the mantelpiece or underneath all the letters for bills that haven’t been paid, and then what do you do about those?"

(Trial co-ordinator/research nurse, practice 1, interview)

Their concern about inequitable access was also raised during training sessions. Through their knowledge of the local population, they raised concerns that this intervention might not work well for those in remote rural areas without mobile phone reception.

"B chimed in quickly that some of their patients were quite remote so [mobile reception] might be an issue."

(Practice managing partner, SIV, practice 3, observation field notes)

In contrast, proactive concerns about or experience in dealing with inequalities were not raised by the centralised administrators. However, once prompted to consider this in interviews, they oriented quickly to think of reasonable solutions.

"…you’ve probably just got to have the…the information in different languages. It’s about accessibility, isn’t it? And, if you are going to offer a face-to-face, you need, obviously, someone who can either interpret on the phone."

(Experienced senior centralised administrator 2, interview)

### Availability of resources

Practice staff were most concerned about the resources required to run screening in primary care due to competing demands on their time from high workloads. Despite being motivated, there was concern aspects of their roles would suffer without extra resource.

"We could do with more staff now, we could do with more clinic time, but that’s not going to happen, being realistic, so it would be a case of managing it in addition to everything else that we’re doing currently… something’s going to have to give a little bit, I guess."

(Clinical nurse 2, practice 3, interview)

Indeed, resource constraints were the most common concern raised by practice staff during training sessions.

"She said that screening 95 participants will be a struggle within the timeframe given, and she asked if they could get the screening invites out today."

(Trial co-ordinator/research nurse, screening training, practice 1, observation field notes)

Practice staff were also conscious of the resource pressures brought by an intervention on other parts of the healthcare system.

“[GP 1, practice 3] there might be a need for the facilities for more echocardiograms”.

(SIV, practice 3, field notes).

When asked, it was suggested that funding could drive motivation and performance by using well known performance-related funding systems, such as the Quality and Outcomes Framework (QOF). This would *“mak[e] sure that it’s important for the surgery… because then that helps pay people”* (trial co-ordinator and research nurse, practice 1, interview).

The centralised administrators, on the other hand, complained less about staff time and even had some flexibility to space out their workload. It might be that having more time available for the programme allowed them to aim for higher performance. Indeed, the different expectations were reflected in training.

"[experienced senior centralised administrator 1] emphasised that it was important to get the right technique… this point was not emphasised to [practice 1] in the screening training."

(centralised administrator training part 2, field notes)

### Scalability

Centralised administrators reported that it would be efficient for them to pool resources so that they can be allocated to where they were needed (as opposed to smaller amounts of resource being stored in primary care practices). They noted that in a centralised system, one would *“have control of devices to spread them among practices as required”* (screening training, site 13, pilot phase, field notes). This could improve the efficiency of a programme. Other aspects of scalability relate to resources and ability to quality assure, discussed previously.

### Standalone versus joined-up care

Centralised administrators did not have a pre-existing system or responsibilities for other aspects of care. This could be an advantage in that the system could *“evolve[e] to work around processes*” (experienced trial staff member1, centralised administrator training 3, TEF). However, a new system would of course have resource implications.

Practices, on the other hand, have the advantage of having a pre-existing IT system in the form of electronic medical records *“where they’ve then got everyone’s contact details and appointment schedules”* (new senior centralised administrator, interview). Such systems also have recall and reminder systems and staff accustomed to their operations.

"…you just give that to the recall people and they add it to their screening programmes, like you get your smears ones and your mammograms…."

(Trial co-ordinator/research nurse, practice 1, interview)

General practices also see patients for other reasons, giving them an opportunity to *“piggy… on the back of something else”* (GP 1, practice 1, interview) and increase screening uptake. To circumvent the concern about a lack of time, these opportunities could be used to simply sign-post patients to a centralised system.

As a result of these other health encounters, practice staff also have pre-existing relationships with patients. This feature can help with credibility, which might improve engagement with a screening programme.

"I would go for the practice… people come in to us and… they do trust the information that we provide."

(Receptionist, practice 1, interview)

Continuity was also valued by practice staff.

"I think that’s probably my personal motivations. I enjoyed doing the screening, it was actually quite nice to have the kind of follow through and do the screening then see the results coming through."

(Trial co-ordinator, practice 2, interview)

However, it was accepted that continuity was not essential for high uptake of screening as *“[the centralised administrators have] shown that [they] can get thousands of patients engaged and screened”* (experienced senior centralised administrator 2, interview).

**Fig. 2:**
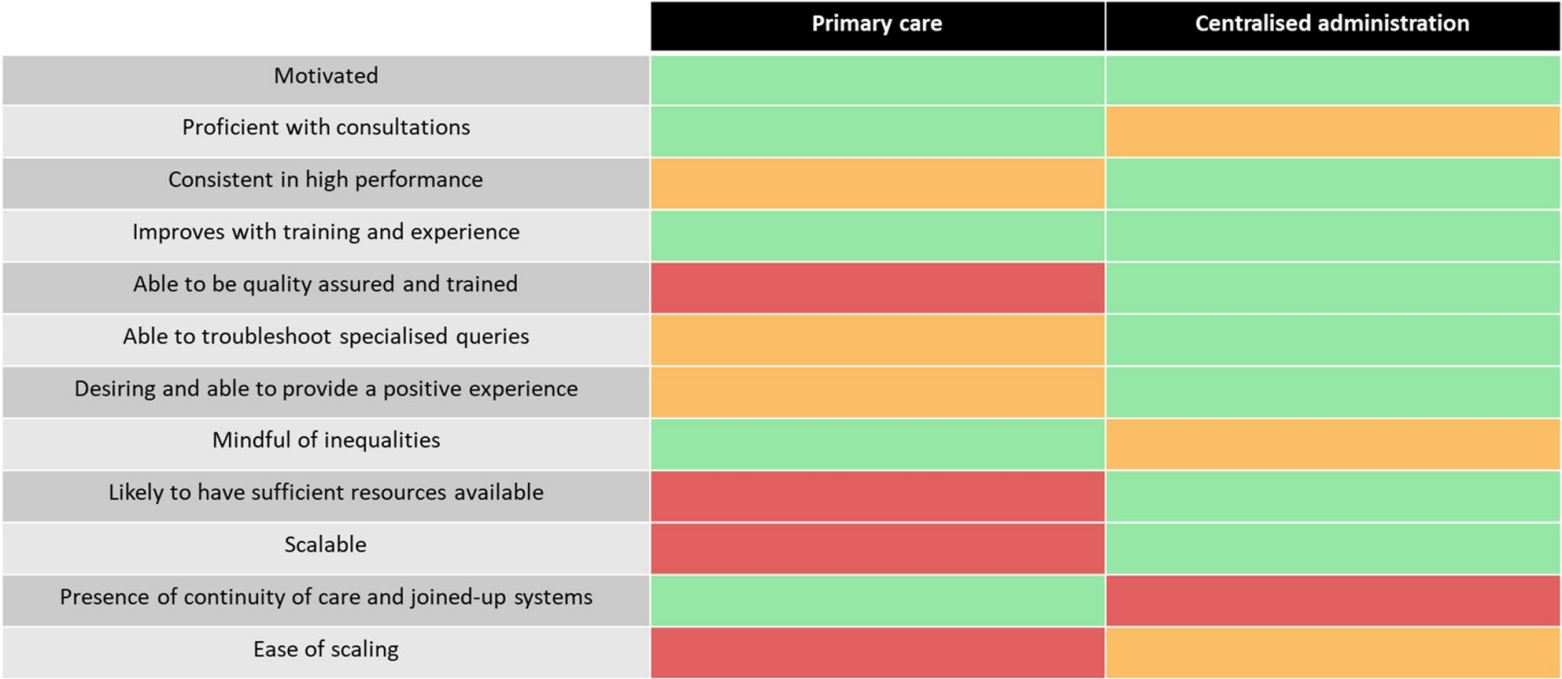
Summary of the relative strengths and weaknesses of primary care and a centralised administration in delivering screening for atrial fibrillation. Adapted from Modi 2023 [13]. Red: weakness. Amber: neither strength nor weakness. Green: strength

The strengths and weaknesses of practice staff and centralised administrators that we identified, in relation to the themes that we developed, are summarised in Fig. 2.

## Discussion

### Summary

Our previous quantitative study showed that there were no significant differences between primary care and a centralised administration in the quality and quantity of ECGs produced in an AF screening programme using hand-held ECG devices [7]. However, in this qualitative study we identified key differences between primary care and a centralised administration.

Primary care staff have valuable experience: notably in consultation skills, experience of their patients, and in dealing with people who experience inequalities in health. These attributes might help them communicate complex and sensitive issues in AF screening with minimal training, join up screening with other care, whilst also being conscious to reduce inequalities in how screening is implemented. They are generally motivated for new interventions that might bring benefit to their patients but they are limited by resources – both time for staff and staff themselves.

A centralised administration would also be motivated to deliver AF screening when supervised by supportive management. They might be more able to provide a consistently high performance in terms of producing high quality screening from an eligible population. Although not initially as skilled as primary care staff with respect to handling patients, with experience and training, they could be proficient in engaging a range of participants, offering a positive and flexible experience to them, and with less pressure of competing workloads. With this their sole workload, they might be more able to be trained in more complex issues related to screening, and be more conveniently quality assured through central mechanisms even when screening is implemented at scale.

A possible solution that capitalises on the strengths of each approach might be a centralised programme that is quality assured and consistent, but that can be signposted to by general practice, who might be in more regular contact with underserved communities.

### Comparison with existing literature

Large scale screening programmes involve multiple groups of staff from different parts of the health system. Choosing the correct location for the mainstay of work is vital for an intervention to be effective, efficient and sustainable in a health system with stretched resources [26]. Despite this, our study is rare in exploring this dimension of an intervention, and doing so with qualitative methods. Similar trials of AF screening e.g. STROKESTOP [27] or LOOP [28] did not compare different models of delivery. We have drawn comparisons with other national screening programmes due to the pragmatic nature of the trial.

We found that the practice-led arm might be successful in engaging their population, including those who are typically underserved by healthcare. With respect to screening programmes, there are no relevant studies in the literature to which this finding can be compared. If the UK cervical cancer screening (practice-led) and bowel cancer screening via postal stool sample (centralised administrator-led) programmes are compared, their uptakes are fairly similar[29], which might suggest that involving the primary care does not improve uptake. However, these programmes are quite different as cervical cancer screening involves the patient attending for an appointment and a rather invasive test being conducted by a health professional. In addition, such comparisons have only been quantitative. In our own study from a quantitative analysis of this SAFER feasibility study, there was no significant difference between practice-led and centralised administrator-led arms with respect to the uptake of screening or the quality and quantity of ECG recordings produced [7]. [30] This also suggests a gap in the literature where quantitative comparisons alone might have missed some nuanced differences. We did not identify any comparisons – quantitative or qualitative - between primary care and centralised administration in screening programmes outside of the UK.

The literature does, however, show that GP endorsement (as opposed to delivery) of screening programmes improves uptake and equity [31–36]. This might suggest that practices do not need to deliver such an intervention – hindered by resources in primary care – but they could simply send invitation letters or signpost patients to a centralised programme. This hybrid approach supported by qualitative studies from other programmes that suggest that primary care staff might not have time to deliver screening interventions as part of usual care [37]. 

We did also note a difference in the power differential between practice staff and patients, compared to centralised administrators and patients. There appeared to be an advantage to centralised administrators trying to flexibly fit around patients’ needs, but it does raise a question about whether GP endorsement works partly because of this power effect [38]. [39] It will therefore be important in screening that involves primary care, to avoid using this power differential to coerce patients to take up screening.

In contrast to the literature that shows a gradient between rural and urban centres partially mediated by socio-economics, access and other factors[40], our data did not attribute differences in delivery based on rurality. This might be explained by the fact that the intervention was remotely delivered which would not discriminate by distance to healthcare.

### Strengths and limitations

This study took place during the Covid-19 pandemic and arguably in a different context. However, workloads are accepted to still be high and remote consulting common in the UK context [8]. As such, careful consideration of whether to use general practice or centralised administration for any new screening programmes remains an important question.

We outline these using Guba and Lincoln’s criteria [41]. By triangulating multiple methods, piloting these methods, and using careful abduction of pre-existing theory[42], we believe this study has reasonable credibility and dependability. However, with the small sample size of three practices, and a single intervention (AF screening), transferability is limited to AF detection programmes, possibly other screening programmes, but not other complex large-scale interventions. On a related point, having only two participants from practice 2, and few participants from certain roles, their views may not have been representative and perspectives might have been missed. However, this study might indicate some dimensions and methodologies to explore in more specific studies of large-scale interventions. In addition, we did find large similarities in findings across practices which suggested sufficient information power in this sample for this research question.Transferability was improved by having three practices that had different characteristics from each other in terms of deprivation and performance scores.

RM was involved in all aspects of the research and this too, could have introduced some bias. By him being part of the research team, although this allowed direct feedback to refine the intervention over the trial, it may have created social desirability bias by participants who wanted to display positivity about the trial, or participatory bias by feeling more compelled to participate. To counter this, it was made clear that participation was voluntary, anonymous and would have no impact on participation in the trial. In addition, questions were phrased to focus on implementation in the NHS rather than issues specific to the practice (see the topic guide, Additional file 2). Openness may have been encouraged by RM’s kinship as a GP and a researcher but this too may have influenced his views from his experience. Whilst these are important lenses through which data can be analysed, the influence of this was minimised by RM keeping a regular reflexive journal of his own thoughts and decisions. In addition, we had regular research meetings to discuss codes and themes amongst the larger research group who were of different disciplines and backgrounds.

Also missing is the patient perspectives on the models. These would be useful avenues of future investigation.

### Implications

This study has implications for the design of AF detection programmes, and possibly other screening programmes. It suggests that there might be trade-offs between efficiency and equity: a cheaper centralised non-clinical administration may be less costly and easier to quality control but might require training and experience to focus on underserved populations. On the other hand, involving general practices might improve equity but would require extra resources. The possibility of a hybrid approach, asking practice staff to signpost and/or endorse an intervention to their patients might be an affordable way to improve equity.

In terms of performance, centralised administrators might provide consistently high-quality and, given time and training, might have adequate communication skills to engage a broad sector of society. Initial investment in centralised administration time, training and a new bespoke IT system might prove cost-effective in the long run.

In terms of research, where there are studies between different models of screening programmes, these should include qualitative methods that allow for emergence of nuanced findings between different approaches. It might also be worth exploring in new or current centralised screening programmes whether hybrid approaches, using primary care to signpost, would improve equitable access to them. In view of the various constraints of primary care, this should involve co-design with relevant stakeholders.

## Conclusions

AF screening programmes and other large-scale screening programmes can be largely based in primary care or in central administration. The choice can be supported by knowing the different strengths and weaknesses of each approach in terms of key issues such as resources available, consistency of high performance, and equity. In the SAFER trial, we adopted centralised administration due to concern about primary care workload and the desire in the context of a trial to have control over the screening process [5]. Policy-makers who intend to increase the detection of AF need to consider these differences with respect to their priorities, resources and concerns about their planned programme, before opting for primary care delivery. A hybrid configuration involving centralised administration (with time to develop), and primary care signposting might be an appropriate model for AF detection programmes and possibly other screening programmes. Where screening programmes are being developed or improved, qualitative methods and co-design might be important to identify and utilise the strengths of various models of delivery.

## Supplementary Information

Supplementary Material 1.

Supplementary Material 2.

Supplementary Material 3.

Supplementary Material 4.

Supplementary Material 5.

## Data Availability

The datasets generated and/or analysed during the current study are not publicly available due to the SAFER trial still being in progress but are available from Andrew Dymond (SAFER@medschl.cam.ac.uk) on reasonable request.

## Abbreviations

AF: Atrial Fibrillation
CFIR: Consolidated Framework for Implementation Research
CHA_2_DS_2_-VASc: Congestive heart failure, Hypertension, Age ≥ 75 years (doubled), Diabetes mellitus, prior Stroke or TIA or thromboembolism (doubled), Vascular disease, Age 65 to 74 years, Sex category (a scoring system with higher scores reflecting a higher risk of stroke in those with atrial fibrillation)
CRN: Clinical Research Network
ECG: Electrocardiogram
GP: General Practitioner
ICF: Informed Consent Form
IT: Information Technology
NIHR: National Institute for Health and Care Research
PIS: Participant Information Sheet
QOF: Quality and Outcomes Framework
SAFER: Screening for Atrial Fibrillation with Electrocardiogram to Reduce Stroke
SIV: Site Initiation Visit
SOP: Standard Operating Procedure
SRQR: Standards for Reporting Qualitative Research
TEF: Training Evaluation Form
UK: United Kingdom

## References

[1] NHS England and NHS Improvement. The NHS long term plan: NHS, 2019. https://www.longtermplan.nhs.uk/wp-content/uploads/2019/08/nhs-long-term-plan-version-1.2.pdf.

[2] NHS England. Newborn and infant physical examination (NIPE) screening programme handbook gov.uk: NHS England. 2024 [Available from: https://www.gov.uk/government/publications/newborn-and-infant-physical-examination-programme-handbook/newborn-and-infant-physical-examination-screening-programme-handbook#nipe-infant-6-to-8-week-screening-examination accessed 30/05/2024.

[3] Public Health England. Bowel cancer screening: programme overview: Public Health England. 2015 [updated 17/03/2021. Available from: https://www.gov.uk/guidance/bowel-cancer-screening-programme-overview accessed 25/08/2021 2021.

[4] Cancer Research UK. About cervical screening: Cancer Research UK; 2022 [updated 18/01/2022. Available from: https://www.cancerresearchuk.org/about-cancer/cervical-cancer/getting-diagnosed/screening/about Accessed 22/01/2023.

[5] Mant J, Modi RN, Dymond A, et al. Randomised controlled trial of population screening for atrial fibrillation in people aged 70 years and over to reduce stroke: protocol for the SAFER trial. BMJ Open. 2024;14(4):e082047. 10.1136/bmjopen-2023-082047. 38670614 10.1136/bmjopen-2023-082047 PMC11057258

[6] Mant J, Modi RN, Charlton P, et al. The feasibility of population screening for paroxysmal atrial fibrillation using handheld ECGs. EP Europace. 2024. 10.1093/europace/euae056. 38411621 10.1093/europace/euae056 PMC10946414

[7] Modi RN, Massou E, Charlton PH, et al. Screening for atrial fibrillation with or without general practice involvement: a controlled study. BMC Prim Care. 2025;26(1):185. 10.1186/s12875-025-02878-y. 40420263 10.1186/s12875-025-02878-y PMC12105410

[8] British Medical Association. Pressures in general practice data analysis 2025 [Available from: https://www.bma.org.uk/advice-and-support/nhs-delivery-and-workforce/pressures/pressures-in-general-practice-data-analysis accessed 20/11/2025.

[9] Lip GY, Nieuwlaat R, Pisters R, et al. Refining clinical risk stratification for predicting stroke and thromboembolism in atrial fibrillation using a novel risk factor-based approach: the Euro heart survey on atrial fibrillation. Chest. 2010;137(2):263–72. 10.1378/chest.09-1584. [published Online First: 2009/09/19]. 19762550 10.1378/chest.09-1584

[10] National Institute for Health and Care Excellence. Atrial fibrillation: diagnosis and management (ng196. National Institute of Health and Care Excellence; 2021.

[11] Office for Health Improvement and Disparities. National General Practice Profiles: GOV.UK. 2022 [Available from: https://fingertips.phe.org.uk/profile/general-practice//page-options/map-ao-4 accessed 27/07/2022.

[12] Malterud K, Siersma VD, Guassora AD. Sample size in qualitative interview studies:guided by information power. Qual Health Res. 2016;26(13):1753–60. 10.1177/1049732315617444. 26613970 10.1177/1049732315617444

[13] Modi R. Delivering a screening programme for atrial fibrillation: a mixed methods investigation. University of Cambridge; 2023.

[14] Damschroder LJ, Aron DC, Keith RE, et al. Fostering implementation of health services research findings into practice: a consolidated framework for advancing implementation science. Implement Science: IS. 2009;4:50. 10.1186/1748-5908-4-50. [published Online First: 2009/08/12]. 19664226 10.1186/1748-5908-4-50 PMC2736161

[15] Saldana JM. The coding manual for qualitative researchers. 3 ed. London, England: SAGE; 2015.

[16] Hammersley M, Atkinson P. Ethnography: principles in practice. Abingdon, UK: Routledge; 2007.

[17] Kirkpatrick DL, Kirkpatrick JD. Evaluating training programmes: the four levels. Third ed. San Francisco, United States: Berrett-Koehler Publishers, Inc.; 2006.

[18] World Health Organization. Evaluating training in WHO. World Health Organization; 2010.

[19] Abramson JH, Abramson ZH. Research methods in community medicine: surveys, epidemiological research, programme evaluation, clinical trials. sixth ed. Chichester, United Kingdom: John Wiley & Sons Ltd.; 2008.

[20] Streiner DL, Norman GR, Cairney J. Health measurement scales: a practical guide to their development and use. fifth ed. New York, United States of America: Oxford University Press; 2015.

[21] Williams R, Brennan J. Collecting and using student feedback on quality and standards of learning and teaching in higher education. Bristol, UK: Centre for Higher Education Research and Information; 2003.

[22] Health Research Authority. Applying a proportionate approach to the process of seeking consent: HRA guidance. England: NHS Health Research Authority; 2017.

[23] Braun V, Clarke V. Using thematic analysis in psychology. Qualitative Res Psychol. 2006;3(2):77–101.

[24] QSR International. NVivo 2020 [Available from: https://www.qsrinternational.com/nvivo-qualitative-data-analysis-software/home accessed 19/06/2020.

[25] O’Brien BC, Harris IB, Beckman TJ, et al. Standards for reporting qualitative research: a synthesis of recommendations. Acad Med. 2014;89(9):1245–51. 10.1097/acm.0000000000000388. [published Online First: 2014/07/01]. 24979285 10.1097/ACM.0000000000000388

[26] Raffle AE, Mackie A, Gray JAM, Screening. Evidence and practice. second ed. Oxford, United Kingdom: Oxford University Press; 2019.

[27] Svennberg E, Engdahl J, Al-Khalili F, et al. Mass screening for untreated atrial fibrillation: the STROKESTOP study. Circulation. 2015;131(25):2176–84. 10.1161/circulationaha.114.014343. [published Online First: 2015/04/26]. 25910800 10.1161/CIRCULATIONAHA.114.014343

[28] Svendsen JH, Diederichsen SZ, Højberg S, et al. Implantable loop recorder detection of atrial fibrillation to prevent stroke (The LOOP Study): a randomised controlled trial. Lancet. 2021. 10.1016/S0140-6736(21)01698-6. 34469766 10.1016/S0140-6736(21)01698-6

[29] Public Health England. PHE screening inequalities strategy. GOV.UK: Public Health England; 2020.

[30] Mant J, Modi RN, Charlton P, et al. The feasibility of population screening for paroxysmal atrial fibrillation using hand-held electrocardiogram devices. EP Europace. 2024;26(3). 10.1093/europace/euae056. 10.1093/europace/euae056 PMC10946414 38411621

[31] Duffy SW, Myles JP, Maroni R, et al. Rapid review of evaluation of interventions to improve participation in cancer screening services. J Med Screen. 2017;24(3):127–45. 10.1177/0969141316664757. 27754937 10.1177/0969141316664757 PMC5542134

[32] Jepson R, Clegg A, Forbes C, et al. The determinants of screening uptake and interventions for increasing uptake: a systematic review. Health Technol Assess (Winchester Eng). 2000;4(14):i–vii. [published Online First: 2000/09/14]. 10984843

[33] Camilloni L, Ferroni E, Cendales BJ, et al. Methods to increase participation in organised screening programs: a systematic review. BMC Public Health. 2013;13(1):464. 10.1186/1471-2458-13-464. 23663511 10.1186/1471-2458-13-464 PMC3686655

[34] Wardle J, von Wagner C, Kralj-Hans I, et al. Effects of evidence-based strategies to reduce the socioeconomic gradient of uptake in the english NHS bowel cancer screening programme (ASCEND): four cluster-randomised controlled trials. Lancet (London England). 2016;387(10020):751–9. 10.1016/s0140-6736(15)01154-x. [published Online First: 2015/12/19]. 26680217 10.1016/S0140-6736(15)01154-X PMC4761689

[35] Zajac IT, Whibley AH, Cole SR, et al. Endorsement by the primary care practitioner consistently improves participation in screening for colorectal cancer: a longitudinal analysis. J Med Screen. 2010;17(1):19–24. 10.1258/jms.2010.009101. 20356941 10.1258/jms.2010.009101

[36] Solutions for Public Health. Systematic review of interventions designed to improve participation in UK National screening programmes amongst underserved population groups – young person and adult screening programmes. gov.uk: Public Health England; 2022.

[37] Sayani A, Vahabi M, O’Brien MA, et al. Perspectives of family physicians towards access to lung cancer screening for individuals living with low income – a qualitative study. BMC Fam Pract. 2021;22(1):10. 10.1186/s12875-020-01354-z. 33413135 10.1186/s12875-020-01354-z PMC7791696

[38] Parsons T. The social system. Glencoe, Ill.: Free Press. 1951:575 pages 22 cm.

[39] Pilnick A, Dingwall R. On the remarkable persistence of asymmetry in doctor/patient interaction: A critical review. Soc Sci Med. 2011;72(8):1374–82. 10.1016/j.socscimed.2011.02.033. 21454003 10.1016/j.socscimed.2011.02.033

[40] Allan R, Williamson P, Kulu H. Unravelling urban–rural health disparities in England. Popul Space Place. 2017;23(8):e2073. 10.1002/psp.2073.

[41] Lincoln YS, Guba EG. Naturalistic inquiry. Beverly hills. Calif : Sage Publications 1985:416 pages : illustrations ; 23 cm.

[42] Kennedy BL. The SAGE handbook of qualitative data collection. London: SAGE Publications Ltd; 2018.

