# Supplementary material for: Implementing screening programmes in primary care versus a centralised administration: a qualitative study of atrial fibrillation screening
Source: BMC Prim Care. 2026 Jan 20;27:60. doi: 10.1186/s12875-026-03172-1 (PMC12903593; doi:10.1186/s12875-026-03172-1)
Supplement: Supplementary file 1 — Supplementary Material 1. [file 12875_2026_3172_MOESM1_ESM.pdf]

<u>Screening appointment checklist</u>

To be used as a checklist during the appointment to ensure you are covering key points.

Key point	Tick (✓) when complete
Ask to speak to or confirm that you are speaking to the participant (using title, first name and surname).	
Confirm that the participant has received the screening pack, including the device?	
Confirm the device ID (found on the back of the device starting with A) matches your record of what you sent them.<br>• Especially if more than one person in the same household is doing the study.	
Confirm that the screening takes 3 weeks:<br>• Requiring the patient to record an ECG 4 times a day.<br>• Each ECG takes 30 seconds.	
Any ECG recorded will be reviewed and a result reported to the GP.<br>This will also mean that the participant has been screened, even if they decide not to continue with the 3 weeks screening.<br><br>We are not clinical and will not be able to review/interpret any ECGs performed during this call.	
Is the participant happy to continue with screening?<br><br>**IMPORTANT! DO NOT ENTER ANY DETAILS ON THE ZENICOR SYSTEM UNLESS THE PARTICIPANT CONFIRMS THEY ARE HAPPY TO CONTINUE TO BE SCREENED**	
If yes, they are still happy to go ahead, set the device up on the Zenicor system ready to record the test ECG.	
If no, participant declines to record an ECG, arrange having the device returned.	
Ask whether they have managed to watch the Zenicor patient instruction video.	
Explain (or reiterate from the video if watched) the key messages for recording an ECG:<br>• be relaxed and sitting down<br>• remain relaxed and still whilst recording<br>• do not move, talk, cough or laugh whilst recording<br>• avoid using hand cream before recording<br>• keep both thumbs in light contact with the electrodes DO NOT PRESS<br>• do not move your thumbs (or fingers) whilst recording<br><br>Advise the participant to put the phone down for a moment to use both hands for the ECG recording and that you will not talk to them whilst they are recording the ECG.	

If no ECG recording/poor quality try reiterating key points but also consider:<br>• using fingers if their hands appear unsteady<br>• query if they have dry skin (progress bar screen doesn't come up and no lights flash – skin not making connection with electrodes)	
If unable to record a good ECG trace after several attempts, explain that it is not always possible to record an ECG in some people and this is no way is a reflection on the participant. Arrange having the device returned.	
The investigation is strictly personal, and the device should not be used or lent to anyone else. *This is also important if there is more than one person in the same household completing screening.*<br>• If more there is more than one person in the same household completing screening, suggest keeping devices in separate rooms or labelling devices with stickers/names.	
If the participant is having difficulty sending the ECGs, tell them not to worry. Ask for them to continue to record ECGs as these will be saved on the device. We recommend that if this is the case, that they do not try to send the ECGs after every recording, as it is this that will drain the batteries.	
Ask whether they may have noticed in the paperwork that there is a tally chart which they can use to help remember how many ECGs they have recorded each day, although this is not compulsory.	
Someone from the team *MAY* contact during the 3 weeks to see how they are getting on. But we won't call everyone so tell them not to worry if they don't hear from us.	
Confirm when the 3 week screening period will finish, however, inform them that someone will be in touch at that point to arrange return of the device. Please ask them **NOT** to return the device **UNTIL** they have heard from us.	
Confirm that if they have any queries relating to the study that they can contact the SAFER Study team (**01223 763491** or safer@medschl.cam.ac.uk), found in the paperwork in their screening pack.	
Confirm that if they have any technical issues with the device they can contact the Zenicor team ([redacted] or [redacted]), found in the paperwork in their screening pack.	
Ask the participant if they have any other questions at this point.	
