# Supplementary material for: Implementing screening programmes in primary care versus a centralised administration: a qualitative study of atrial fibrillation screening
Source: BMC Prim Care. 2026 Jan 20;27:60. doi: 10.1186/s12875-026-03172-1 (PMC12903593; doi:10.1186/s12875-026-03172-1)
Supplement: Supplementary file 2 — Supplementary Material 2. [file 12875_2026_3172_MOESM2_ESM.docx]

Supplementary file 2. Interview topic guide


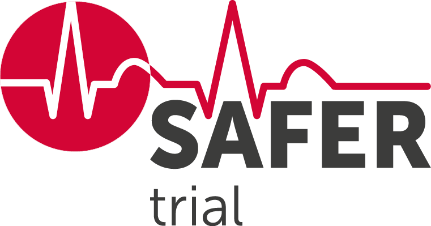

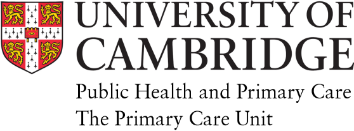


**SAFER Trial practice staff/trial team interview topic guide**

**Introduction and Consent Process**

- Researcher to introduce themselves and check understanding of trial and processes

- Informed consent and permission to audio record the interview obtained from the participant

- Start recording and check that they are happy with consent statements and sign pp noting that verbal consent and recorded in audio file

**A. Your role in AF diagnosis and treatment**

Can you tell me a little bit about your current role?

*Prompt:*

- *General responsibilities*
- *Experiences of or responsibilities for AF diagnosis and treatment*
- *Other cardiovascular or relevant responsibilities*
- *Experience of or responsibilities for delivery of training*
- *(mid and late trial staff interview) has anything changed in your role?*

**B. Training (baseline interviews only)**

*If they have given training:*

I’d like to explore your experiences of training in AF detection and treatment.

(if yes) - What do you think about the training you are providing?

*Prompt:*

- *Appropriate length?*
- *Appropriate time? (relative to screening, and within day/week/month/year etc)*
- *Appropriate format?*
- *Appropriate audience?*
- *Anything you would prefer to do differently?*
- *What are the most typical areas of uncertainty?*
- *Are there particular misunderstandings you need to address?*

*If they have received training:*

I’d like to explore your experiences of training in AF detection and treatment.

What do you think about the training you received?

- *Appropriate length?*
- *Appropriate time? (relative to screening, and within day/week/month/year etc)*
- *Appropriate format?*
- *Anything particularly good about the training?*
- *Anything you particularly learnt from the training?*
- *Anything you would prefer to have been done differently?*
- *What did you want to learn before going into the training?*
- *What are the most typical areas that you were uncertain of even after training?*

**C. Attitudes to AF screening**

As you know, we are currently embarking on a trial of screening for AF in primary care, with the aim of determining whether a national screening programme may be effective and cost effective. What are your thoughts on delivering the AF screening?

*Prompt:*

- *Whether AF screening is worthwhile*
- *How does AF screening compare to other screening in your view?*
- *Whether the health system and population will take to AF screening well.*

**D. For practice staff: Current approaches to AF detection and treatment**

How does your practice usually approach the detection and treatment of AF?

- *What was working?*
- *What was not working?*
- *Did you think you should be doing anything differently?*
- *How do your approaches fit in with other initiatives, such as other local practices, CCG initiatives, and available secondary care circumstances?*
- *Can you tell me if there has been anything that you’ve seen from national bodies like NICE or the government, or local bodies like the CCG or even in the media about AF or its treatment in the recent past? Has that had an impact on you (or your patients)?*
- *(Ensure cover both detection AND treatment)*

**E. Implementation of AF screening**

Next, I’d like to explore a little bit about implementing this screening programme.

*Prompt:*

- *What do you like about the screening programme?*
- *What do you not like about the screening programme?*
- *Who is doing what with regards to the SAFER trial? Who knows about it?*
- *(for trial team) What did you talk about in the call when you arranged to send the device (delivery, mode, pacemaker etc)?*
- *How long did they take? Did you get time scheduled for that? What about the call for device return?*
- *How long were the screening appointments?*
- *(for practice and trial team) Did you do anything to increase uptake of screening or to raise the profile?*
- *(trial team) What are your thoughts about the device and using it?*
- *(GPs and nurses) What about anticoagulation – what are your thoughts on that?*
- *(For GPs/ANPs and trial team conducting screening appts) what do you understand about the screening results?*
- *(For trial team in mid and late interviews) have you had to explain what kind of results will come back from the screening?*
- *(for GPs/ANPs) What do you think about the reports that come back?*
- *(for GPs/ANPs) The traces go through an automatic filter which means it may miss some of the other diagnoses that can be picked up on ECGs – how do you feel about this?*
- *(trial team) Do you think that administrators in a larger national teamwill take on screening well.*
- *How does it fit into your daily routines and workflows?*
- *Do you feel that your role in this study is within your general job description or do you feel uncomfortable with it? How do you feel now, after having done it?*
- *Have you encountered any particular difficulties at this stage?*
- *How are you implementing it – leaders? Audits? – how should you?*
- *On a larger scale, if AF screening became national, do you think that sending the device, arranging the screening consultation and doing the screening consultation would be better to be done by a GP practice, a central administrative body, or even by patients just watching a video or reading a leaflet? Why?*
- *What do you think we can do in this trial to improve the understanding and access by people who speak different languages?*
- *Looking ahead, what do you think the major challenges are for the detection and treatment of AF (either in trial or generally)?*

**F. Remote vs face to face**

What do you think about delivering this remotely versus face to face?

- *Problems?*
- *Benefits?*
- *Strategies to overcome it?*

**G. Other issues to cover**

Can you think of anything else that we have not discussed, about this topic, which you would like to raise?

**Interview close**

- Participants will be thanked for taking part in the trial and reminded that their responses will remain confidential
- Participants will once again have the opportunity to ask the researcher any questions and will also be given the opportunity to raise any additional issues that they feel are relevant to the topic
- Participants will be reminded that they can contact the research team at any time using the details on the information leaflet should they have any further questions or wish to remove their responses from the trial.
