# Supplementary material for: Implementing screening programmes in primary care versus a centralised administration: a qualitative study of atrial fibrillation screening
Source: BMC Prim Care. 2026 Jan 20;27:60. doi: 10.1186/s12875-026-03172-1 (PMC12903593; doi:10.1186/s12875-026-03172-1)
Supplement: Supplementary file 3 — Supplementary Material 3. [file 12875_2026_3172_MOESM3_ESM.docx]

Supplementary file 3. Training evaluation form for screening training for general practice staff


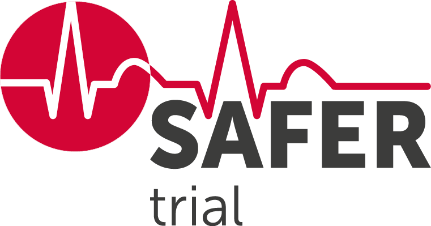

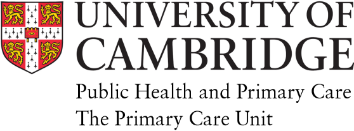


**SAFER Study:**

**Screening for Atrial Fibrillation with ECG to Reduce stroke - feasibility**

**Evaluation of screening training**

In order to evaluate the training today and assess its impact, we would be very grateful if you could complete this evaluation form. It includes a few questions so that we can understand knowledge before and after the training, and a general feedback section. We do not expect you to know the answers to any of the questions prior to receiving the training. Whether you fill it in or not, and what answers you give will have no impact on your role or participation in the study. Your responses will be anonymous, confidential and kept securely.

**Please answer these questions about you:**

1. Practice name:
2. Lead GP on study (PI):
3. Your job title:

**Understanding your knowledge: pre-training**

**For each of the following questions, please tick the SINGLE best answer:**

1. How often and for how long are we advising patients to use the device at home?
2. Once daily for two weeks 🞏
3. Twice daily for four weeks 🞏
4. Three times daily for three weeks 🞏
5. Twice daily for one week 🞏
6. Four times daily for three weeks 🞏
7. Which of the following is an exclusion criteria for the SAFER study?
8. High bleeding risk 🞏
9. Having a pacemaker 🞏
10. On anticoagulation 🞏
11. Those with known AF 🞏
12. On an anti-platelet 🞏
13. What is needed for transmission of the ECG reading from the device?
14. GPS signal 🞏
15. Wifi signal 🞏
16. Radio-wave signal 🞏
17. Mobile signal 🞏
18. Wired connection 🞏
19. What is the minimum number of dots of battery life needed to cover a patient for performing measurements and transmitting ECGs at home?
20. 5 🞏
21. 4 🞏
22. 3 🞏
23. 2 🞏
24. 1 🞏
25. When the patient attends for a screening appointment, what type of consent is needed?
26. Written 🞏
27. None 🞏
28. Electronic signature 🞏
29. Written and witnessed 🞏
30. Verbal 🞏

**Please only complete the following section AFTER completing your training**

**Training feedback**

**To what extent do you agree with the following statements? Please circle the most appropriate number:**

| **Course** | | | | | |
| --- | --- | --- | --- | --- | --- |
|  | Strongly disagree | Disagree | Neutral | Agree | Strongly agree |
| The learning objectives were achieved | 1 | 2 | 3 | 4 | 5 |
| The training was relevant to what we need to know | 1 | 2 | 3 | 4 | 5 |
| I feel like I could start to perform my role in the SAFER study | 1 | 2 | 3 | 4 | 5 |
| There was the correct amount of content for the time given | 1 | 2 | 3 | 4 | 5 |
| **Trainer(s)** | | | | | |
| The trainer knew the topic well | 1 | 2 | 3 | 4 | 5 |
| The trainer was stimulating | 1 | 2 | 3 | 4 | 5 |
| I felt that I could ask questions if I wanted to | 1 | 2 | 3 | 4 | 5 |
| The trainer responded to questions well | 1 | 2 | 3 | 4 | 5 |

Please provide any other comments on the training:

**Understanding your knowledge: post-training**

**For each of the following questions, please tick the SINGLE best answer:**

1. How often and for how long are we advising patients to use the device at home?
2. Twice daily for one week 🞏
3. Twice daily for four weeks 🞏
4. Four times daily for three weeks 🞏
5. Once daily for two weeks 🞏
6. Three times daily for three weeks 🞏
7. Which of the following is an exclusion criteria for the SAFER study?
8. On anticoagulation 🞏
9. High bleeding risk 🞏
10. Having a pacemaker 🞏
11. On an anti-platelet 🞏
12. Those with known AF 🞏
13. What is needed for transmission of the ECG reading from the device?
14. Mobile signal 🞏
15. GPS signal 🞏
16. Wifi signal 🞏
17. Wired connection 🞏
18. Radio-wave signal 🞏
19. What is the minimum number of dots of battery life needed to cover a patient for performing measurements and transmitting ECGs at home?
20. 1 🞏
21. 2 🞏
22. 3 🞏
23. 4 🞏
24. 5 🞏
25. When the patient attends for a screening appointment, what type of consent is needed?
26. Written 🞏
27. Written and witnessed 🞏
28. Verbal 🞏
29. Electronic signature 🞏
30. None 🞏

Thank you very much for your time and effort in completing this form. Should you have any further questions, concerns or complaints please contact the SAFER team:

Tel: **01223 763491** (Monday to Friday, 9am – 5pm)

Email: [safer@medschl.cam.ac.uk](mailto:safer@medschl.cam.ac.uk)

The SAFER Study
Primary Care Unit
Department of Public Health and Primary Care
University of Cambridge
Strangeways Research Laboratory
Worts’ Causeway
Cambridge CB1 8RN
