# Supplementary material for: Implementing screening programmes in primary care versus a centralised administration: a qualitative study of atrial fibrillation screening
Source: BMC Prim Care. 2026 Jan 20;27:60. doi: 10.1186/s12875-026-03172-1 (PMC12903593; doi:10.1186/s12875-026-03172-1)
Supplement: Supplementary file 4 — Supplementary Material 4. [file 12875_2026_3172_MOESM4_ESM.docx]

Supplementary file 4. Training evaluation form for trial team training


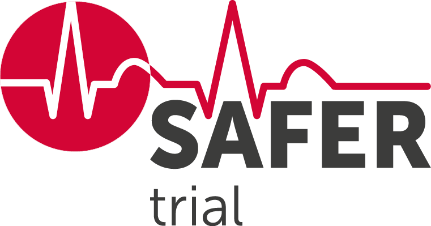

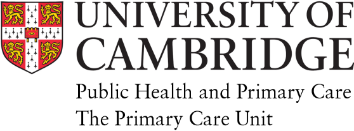


**SAFER Trial:**

**Screening for Atrial Fibrillation with ECG to Reduce stroke**

**Evaluation of training for trial management team**

In order to evaluate the training today so that we can improve it and assess what impact it has had on the SAFER trial, we would kindly ask you to complete this evaluation form. The answers you give will have no impact on your role or participation in the trial.

**Please answer these questions about you:**

**Role:**

**Training feedback**

**To what extent do you agree with the following statements? Please circle the most appropriate number. 1 = strongly disagree. 5 = strongly agree.**

| **Course** | | | | | |
| --- | --- | --- | --- | --- | --- |
|  | **Strongly disagree** | **Disagree** | **+/-** | **Agree** | **Strongly agree** |
| The learning objectives were achieved | 1 | 2 | 3 | 4 | 5 |
| The training was relevant to what I need to know | 1 | 2 | 3 | 4 | 5 |
| I feel like I could start to perform my role in the SAFER trial | 1 | 2 | 3 | 4 | 5 |
| There was the correct amount of content for the time given | 1 | 2 | 3 | 4 | 5 |

**Please provide some brief comments to help us improve the training of future administrators:**

**Conducting screening appointments**

1. **What was most useful about the training session with regards to preparing you to undertake screening appointments?**
2. **What could have been done to improve your preparation to undertake screening appointments?**
3. **Is there anything that you still feel unprepared for or concerned about, with regards to undertaking screening appointments? If so, what?**

**Dealing with ECGs**

1. **What was most useful about the training session with regards to preparing you for managing the ECG readings?**
2. **What could have been done to improve your preparation to manage the ECG readings?**
3. **Is there anything you still feel unprepared for or concerned about, with regards to managing the ECG readings? If so, what?**

Thank you very much for your time and effort in completing this form. Should you have any further questions, concerns or complaints about the research please contact Rakesh Modi ([rnm30@medschl.cam.ac.uk](mailto:rnm30@medschl.cam.ac.uk)).
